# Intra-articular calcaneal fracture: closed reduction and balloon-assisted augmentation with calcium phosphate cement: a case report

**DOI:** 10.1186/1757-1626-2-9290

**Published:** 2009-12-09

**Authors:** Artan Bano, Dritan Pasku, Apostolos Karantanas, Kalliopi Alpantaki, Xenia Souvatzis, Pavlos Katonis

**Affiliations:** 1 Department of Orthopaedic and Traumatology, University Hospital of Heraklion, T.K.: 71003 Voutes, Heraklion, Crete, Greece; 2 Department of Radiology, University Hospital of Heraklion, T.K.: 71003 Voutes, Heraklion, Crete, Greece; 3 Department of Anaesthesiology, University Hospital of Heraklion, T.K.: 71003 Voutes, Heraklion, Crete, Greece

## Abstract

**Introduction:**

For decades, open reduction and internal fixation was the surgical treatment of choice for intra-articular calcaneal fractures, either with or without any augmentation. Delayed weight bearing and wound-related complications are still unresolved. Aiming at a minimally invasive therapy with accelerated mobilization, we applied closed reduction and balloon-assisted augmentation with calcium phosphate cement.

**Case presentation:**

A 45-years-old Greek man with intra-articular calcaneal fracture was treated with closed reduction and balloon assisted augmentation with calcium phosphate cement. Follow-up was performed using the Maryland foot score, plain radiographs and multidirectional computerized tomography. Early full weight-bearing was performed at the end of the first week postoperatively. There was no need for secondary reconstructive procedures at the 2 year follow-up. The patient had minimal problems regarding the pain, subtalar motion and peroneal impingement. There was no significant further collapse of the subtalar calcaneal articular surface radiologically.

**Conclusion:**

The closed reduction and balloon assisted augmentation with calcium phosphate cement of intra-articular calcaneal fractures is a minimally invasive surgical procedure which led to early full weight bearing, good functional patient outcomes and a low complication rate.

## Introduction

The complex morphology and the joint deformity of the intra-articular calcaneal fractures (IACF) have been classified using the computerized tomography (CT) findings [1]. Despite the improvement in understanding and classifying the IACF, the open reduction with or without augmentation remained the surgical procedure of choice. Its main limitations include delayed weight bearing and wound-related complications [2-4].

Aiming at a minimally invasive surgery and early mobilization, we present the surgical technique and the clinical course in a patient with IACF who underwent closed reduction and balloon assisted augmentation with calcium phosphate (Calcibon, Biomet, Warsaw, IN) cement (CPC).

## Case presentation

**Figure 1:**
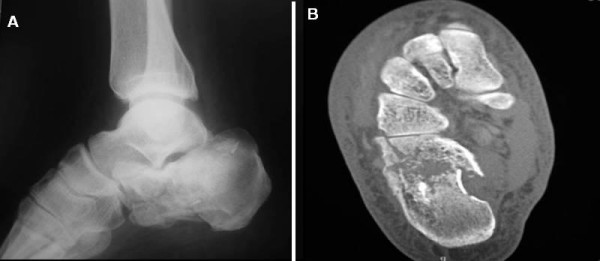
**a) Preoperative lateral radiograph and (b) transverse plane CT scan demonstrating calcaneal fracture with intra-articular displacement**.

A 45-year-old Greek man with IACF was prospectively evaluated. The patient presented with right closed IACF in March 2007 at our Orthopaedic and Traumatology Department. The treatment consisted of a closed reduction technique combined with balloon-assisted fracture augmentation with CPC. The mechanism of trauma was axial loading. Standard radiographic evaluation and MDCT scans were performed preoperatively (Figure 1a and Figure 1b). The calcaneal fracture was classified as a type III according to the Sanders classification [1].

### Operative procedure

**Figure 2:**
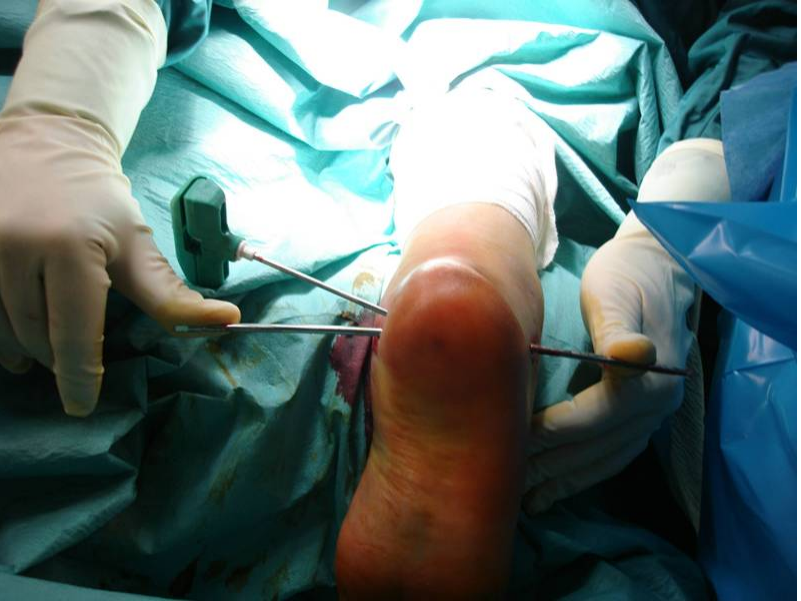
**A traction wire is placed to correct calcaneal malalignment**.

**Figure 3:**
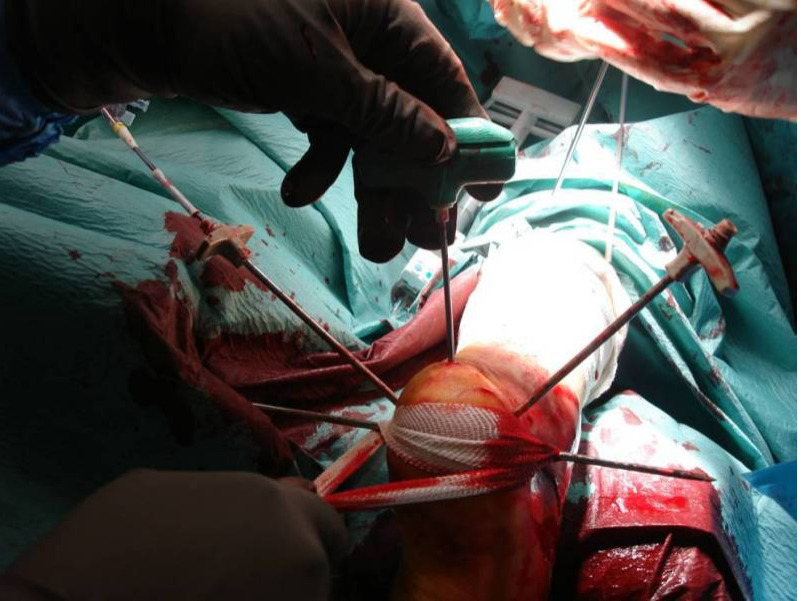
**A guide pin and cannulas are placed under biplanar fluoroscopy guidance to augment the calcaneal fracture**.

**Figure 4:**
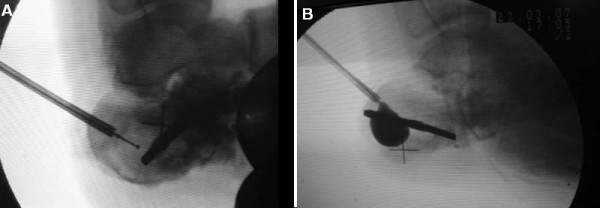
**Intraoperative view demonstrating the position of the balloon (a) before and (b) after inflation**.

A calcaneus traction wire was placed from the posterolateral area of the calcaneus perpendicular to the longitudinal axis of the calcaneus and the varus, valgus malalignment was corrected (Figure 2). Fluoroscopy was used to determine the quality of the calcaneal alignment and the degree of fracture reduction. A stylet and cannula were placed into the calcaneus followed by insertion of a bone tamp attached to a digital manometer (Kyphon^®^, Medtronic, Minneapolis, MN, USA) (Figure 3). The balloon was inflated gradually under fluoroscopy (Figure 4a and Figure 4b). At this time the bone cement was prepared immediately prior to its injection into the defect and the balloon was removed. The fracture void was cleaned of blood with NS solution and a bone filler device was inserted through the cannula to augment the calcaneal body. No arrest of blood supply was needed and no cast was applied.

Passive ankle ROM was applied at the end of the procedure. The duration of surgery was 20 min. The patient showed immediate pain relief from the 1^st ^postoperative day. Limb elevation and foot pump were used to prevent ankle swelling.

**Figure 5:**
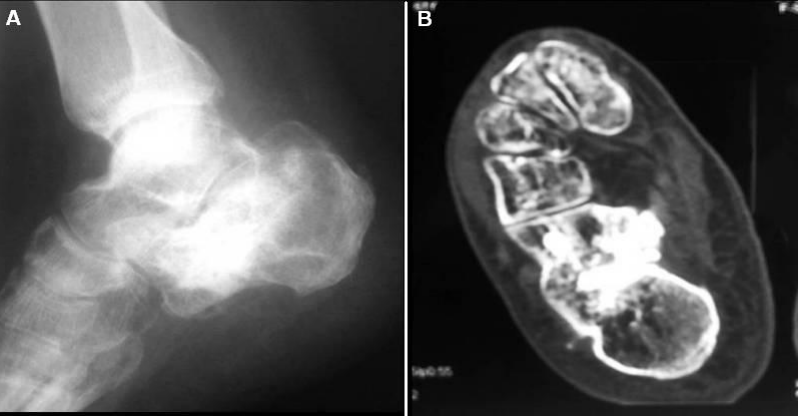
**Two years postoperative (a) lateral radiograph and (b) CT scan demonstrating no loss of reduction**.

Clinical follow-up was performed 6 to 24 months postoperatively, using the Maryland foot score for the calcaneal fractures [1]. Postoperative MDCT scan was obtained for the evaluation of the cement position, the integrity of the joint surface and the calcaneal alignment. Active and passive motion exercises were performed under physiotherapeutic support. The patient was able to walk with partial weight-bearing beginning on second day. Full weight-bearing was performed after the first week postoperatively when the ankle swelling decreased. No superficial or deep infections were recorded. There was no need to undergo a secondary reconstruction procedure. Bone healing was seen on plain radiographs at 12 weeks. The immediate postoperative radiographs and CT scan revealed no loss of fracture reduction and good calcaneal alignment. Complete filling of the bone defects was seen. Radiography and CT scan evaluation at 2 year follow-up showed good fracture alignment (Figure 5a and Figure 5b). No significant difficulty in toe-walking, heel walking or impingement of the peroneal tendons was observed.

## Discussion

Restoration of the mechanical stability for earlier full weight-bearing and patient mobilization with minimal morbidity and complications, were our surgical treatment goals. The principal aims of surgical treatment include restoration of height, length, width, and axis with anatomical reconstruction of all joint surfaces and restoration of function by primary stable osteosynthesis. Options include open reduction combined with internal fixation (ORIF) with or without grafting or bone cement augmentation, wire circular frame procedure, open reduction and arthroscopically assisted internal fixation and primary subtalar arthrodesis [1,3-7]. Several minimally invasive procedures have been described for treating calcaneal fractures: intraoperative CT-guided correction, closed reduction combined with percutaneous screw fixation and the Essex-Lopresti reduction [8-10]. Soft tissue protection is important in surgical management of the calcaneus. Folk et al. in their study including operative treatment of 190 calcaneal fractures, showed that in 25% of cases there was some form of wound complication and 21% of these required surgical treatment [2]. Skin necrosis or infections are not uncommon. Zwipp et al., who studied 123 cases treated with osteosynthesis for displaced IACF, reported an incidence of 8.3% superficial wound edge necrosis [11]. The ORIF of IACF is associated with partial weight-bearing for 6 to 12 weeks postoperatively, depending on the severity of the fracture. Earlier full weight-bearing was performed in IACF treated with ORIF and augmentation with calcium bone cement [12]. Autogenous bone from the iliac crest remains the preferred bone graft for filling void fractures but this procedure is associated with donor-site morbidity and major complications. An alternative to autogenous bone grafts are synthetic bone materials such as CPC. Bajammal et al in a meta-analysis of fourteen randomized controlled trials suggested that the use of CPC for the treatment of fractures in adult patients is associated with a lower incidence of pain and a decreased risk of fracture reduction loss [13]. The use of CPC improved the stability of calcaneal fracture constructs, allowing earlier weight-bearing without loss of reduction. Thordarson et al. demonstrated a significant increase in the compressive fatigue strength of an in vitro calcaneal fracture-repair construct that included filling of the entire defect with SRS bone cement [14]. The compressive strength of calcium phosphate bone cement has been documented to be fifty-five megapascals, which is equivalent to the strength of intact cancellous bone [15].

In our presented case no development of any soft tissue inflammatory response was observed. The patient was examined during the postoperative follow-up at 6 weeks, 6, 12, 24 months and did not show any loss of correction on plain radiographs.

The stabilization of calcaneal fracture fragments with CPC eliminates painful micromotions and thus may contribute to pain relief by means of stable osteosynthesis.

We consider that the closed reduction and fracture augmentation with CPC without ORIF, can restore the mechanical stability and prevent further collapse of the subtalar calcaneal articular surface, leading to quicker rehabilitation, a shorter period of disability and minimal complications in patients with closed and without extended community of articular surfaces. The patient reported total relief of pain level from the first postoperative day. Larger series and longer follow-up are required to assess prospectively the value of the above applied technique.

## Conclusion

The balloon-assisted reduction of IACF and the augmentation with CPC is a minimally invasive and short surgical procedure with minor morbidity. Early postoperative weight bearing and mobilization were possible in our patient who also demonstrated a clinically and radiographically acceptable outcome at the 2 year follow-up. Larger series and correlations with standard techniques are needed to assess the impact of this minimally invasive treatment on the final functional outcome.

## Abbreviations

CPC: calcium phosphate cement; IACF: intra-articular calcaneal fracture; MDCT: multidirectional computerized tomography; NS: normal saline; ORIF: open reduction and internal fixation; ROM: range of motion; SRS: skeletal repair system.

## Consent

Written informed consent was obtained from the patient for publication of this case report and accompanying images. A copy of the written consent is available for review by the journal's Editor-in-Chief.

## Competing interests

The authors declare that they have no competing interests.

## Authors' contributions

AB contributed to the preparation of the manuscript and performed the surgical procedure. DP contributed to the preparation of the manuscript and performed the surgical procedure. AK prepared the final manuscript. KA conducted a literature search. XS performed the anaesthesiology for the operative procedure. AB, DP and PK contributed to the postoperative management and the follow-up of the patient. All authors reed and approved the final manuscript.

## References

[B1] Sanders R Fortin P DiPasquale T Walling A Operative treatment in 120 displaced intraarticular calcaneal fractures Clin Orthop 1993 290 87 95 8472475

[B2] Folk JW Starr AJ Early JS Early wound complications of operative treatment of calcaneus fractures: analysis of 190 fractures J Orthop Trauma 1999 13 369 372 10.1097/00005131-199906000-00008 10406705

[B3] Zmurko MG Karges DE Functional Outcome of Patients Following Open Reduction lnternal Fixation for Bilateral Caicaneus Fractures Foot Ankle Int 23 917 21 12398143 10.1177/107110070202301005

[B4] Zwipp H Rammelt S Barthel S Calcaneal fractures - open reduction and internal fixation (ORIF) Injury 2004 35 S-B46-S-B54 10.1016/j.injury.2004.07.011 15315878

[B5] Sarkar MR Stahl J Wachter N Schwamborn M Schnettler RWW Kinzl L Defect Reconstruction in Articular Calcaneus Fractures with a Novel Calcium Phosphate Cement Eur J Trauma 2002 28 340 348 10.1007/s00068-002-1179-y

[B6] Gavlik JM Rammelt S Zwipp H The use of subtalar arthroscopy in open reduction and internal fixation of intra-articular calcaneal fractures Injury 2002 33 63 71 10.1016/S0020-1383(01)00077-8 11879836

[B7] Mauffrey C Klutts P Seligson D The use of circular fine wire frames for the treatment of displaced intra-articular calcaneal fractures J Orthopaed Traumatol 2009 10 9 15 10.1007/s10195-008-0037-z PMC2657348 19384629

[B8] Mayr E Hauser H Ruter A Bohndorf K Minimally invasive intraoperative CT-guided correction of calcaneal osteosynthesis Unfallchirurg 1999 102 239 244 10.1007/s001130050398 10232042

[B9] Tornetta P Percutaneous treatment of calcaneal fractures Clin Orthop Relat Res 2000 375 91 96 10.1097/00003086-200006000-00011 10853157

[B10] Tornetta P The Essex-Lopresti reduction for calcaneal fractures revisited J Orthop Trauma 1998 12 469 473 10.1097/00005131-199809000-00007 9781770

[B11] Zwipp H Tscherne H Thermann H Weber T Osteosynthesis of displaced intraarticular fractures of the calcaneus. Results in 123 cases Clin Orthop 1993 290 36 40 8472474

[B12] Thordarson DB Bollinger M SRS Cancellous Bone Cement Augmentation of Calcaneal Fracture Fixation Foot & Ankle Int 2005 26 347 352 15913516 10.1177/107110070502600501

[B13] Bajammal SS Zlowodzki M Lelwica A Tornetta P Einhorn TA Buckley R Leighton R Russell TA Larsson S Bhandari M The Use of Calcium Phosphate Bone Cement in Fracture Treatment. A Meta-Analysis of Randomized Trials J Bone Joint Surg Am 2008 90 1186 1196 10.2106/JBJS.G.00241 18519310

[B14] Thordarson DB Hedman TP Yetkinler DN Eskander E Lawrence TN Poser RD Superior Compressive Strength of a Calcaneal Fracture Construct Augmented with Remodelable Cancellous Bone Cement J Bone Joint Surg Am 1999 81 239 246 10073587 10.2106/00004623-199902000-00011

[B15] Rohl L Larsen E Linde F Odgaard A Jorgensen J Tensile and compressive properties of cancellous bone J Biomech 1991 24 1143 1149 10.1016/0021-9290(91)90006-9 1769979

